# Transcutaneous Auricular Vagal Nerve Stimulation and Disorders of Consciousness: A Hypothesis for Mechanisms of Action

**DOI:** 10.3389/fneur.2020.00933

**Published:** 2020-08-25

**Authors:** Marie-Michele Briand, Olivia Gosseries, Bernard Staumont, Steven Laureys, Aurore Thibaut

**Affiliations:** ^1^Coma Science Group, GIGA Consciousness, University of Liège, Liège, Belgium; ^2^Centre du Cerveau^*2*^, University Hospital of Liège, Liège, Belgium; ^3^Physical Medicine and Rehabilitation Department, Institut de Réadaptation en Déficience Physique de Québec, Quebec City, QC, Canada

**Keywords:** disorders of consciousness, post-coma, transcutaneous auricular vagal nerve stimulation, brain injury, non-invasive brain stimulation, brain network, functional magnetic resonance imaging

## Abstract

Disorders of consciousness (DoC) are the hallmark of severe acquired brain injuries characterized by abnormal activity in important brain areas and disruption within and between brain networks. As DoC's therapeutic arsenal is limited, new potential therapies such as transcutaneous auricular vagal nerve stimulation (taVNS) have recently been explored. The potential of taVNS in the process of consciousness recovery has been highlighted in recent studies with DoC patients. However, it is not clear how taVNS plays a role in the recovery of consciousness. In this article, we first describe the neural correlates of consciousness, the vagus nerve anatomy and functions, along with the results of functional magnetic resonance imaging studies using taVNS. Based on consciousness recovery and taVNS mechanisms, we propose the Vagal Cortical Pathways model. This model highlights four consecutive pathways (A. Lower brainstem activation, B. Upper brainstem activation, C. Norepinephrine pathway, and D. Serotonin pathway) likely to have an impact on patients with a brain injury and DoC. Additionally, we suggest six different mechanisms of action: (1) Activation of the ascending reticular activating system; (2) Activation of the thalamus; (3) Re-establishment of the cortico-striatal-thalamic-cortical loop; (4) Promotion of negative connectivity between external and default mode networks by the activation of the salience network; (5) Increase in activity and connectivity within the external network through the norepinephrine pathway; and (6) Increase in activity within the default mode network through the serotonin pathway. This model aims to explain the potential therapeutic effects that taVNS has on brain activity in the process of consciousness recovery.

## Introduction

Acquired brain injury is an umbrella term for any brain damage that occurs after birth and that is not hereditary, congenital, degenerative, or induced by birth trauma (1). The mechanisms leading to acquired brain injury include infection, anoxia and traumatic brain injuries, which can induce global or focal injury pathophysiology (e.g., cerebral hematoma, contusion, diffuse axonal injury). All of these etiologies have the potential to generate disorders of consciousness (DoC), especially since advanced medical care has improved survivor rate after severe brain injuries (2).

DoC patients represent a clinical challenge because of the difficulty of diagnosis [coma, unresponsive wakefulness syndrome—UWS (3), and minimally conscious state—MCS (4)] as, to date, no single assessment can lead to a definite diagnosis. First, up to 40% of clinical misdiagnosis has been reported when an appropriate and validated scale is not used (5, 6). Advanced technologies and analytic approaches, such as high-density electroencephalography (EEG), functional magnetic resonance imaging (fMRI), and positron emission tomography (PET), represent tools used to measure the level of consciousness in patients with DoC (7), but none of them are able to ascertain the level of consciousness. Consequently, diagnosis is often made by combining clinical, neurophysiological and neuroimaging evaluations in the hope of finding a consistent pattern among the techniques used while being as accurate as possible. Nonetheless, inconsistency is still a possibility (8).

Consciousness has been described as a combination of two components: arousal and awareness (9). Arousal refers to the degree of alertness or vigilance while awareness refers to the capacity to interact with the environment or the self (10). Coma is defined as the absence of both arousal and awareness. UWS [previously called vegetative state (11)] is defined by the absence of awareness of the environment or of the self, observed at the bedside, despite the presence of intermittent periods of wakefulness (arousal), either spontaneously or following tactile, auditory or painful stimulations (3). MCS is characterized by variations in arousal levels and recovery of fluctuant, but reproducible signs of awareness such as visual pursuit, object localization, or command following (4). In this case, although awareness is fluctuating, remnants of willful behaviors are present. While several behavioral scales have been developed, the Coma Recovery Scale-Revised [CRS-R—(12)] is currently the recommended scale for categorizing the level of consciousness (13).

Over the last few years, a growing interest has been observed in the development of therapeutic strategies to improve and stimulate the cognitive and functional recovery of DoC patients, both in the acute and prolonged states (14). One of the emerging treatments is the vagal nerve stimulation (VNS). After the report of clinical improvements following the use of VNS devices in four articles [(15–18); see more details in sections Unique Case of an Implanted Vagal Nerve Stimulation in a Patient With DoC for VNS study and Studies in Patients With DoC for taVNS studies, respectively], we here aim to explain the possible mechanisms of action underlying transcutaneous auricular VNS (taVNS) in DoC: (i) by presenting a model that describes the mechanisms of action by which taVNS can modulate brain activity; and (ii) by evaluating its potential role and efficacy in the processes of consciousness recovery of brain injured patients.

An overview of the most described neural correlates of consciousness and the vagal nerve anatomy and functions is first presented to facilitate the understanding of the model proposition and the mechanisms of action of taVNS in consciousness recovery processes. We then describe the current state-of-the-art for taVNS and its impact on brain activity in healthy subjects and DoC patients. Then, the Vagal Cortical Pathways model, i.e., our hypothesis about taVNS general mechanisms of action on the brain, is described and challenged by taVNS neuroimagery studies. Finally, when applying this model to patients with DoC, six mechanisms of action are proposed to explain the potential effects on consciousness recovery.

## Neural Correlates of Consciousness

Scientists have been searching for key structures of consciousness. Our intention is to provide a comprehensive overview of the main structures and networks involved in consciousness processes and recovery.

### Key Brain Areas

Three important structures have been described as cornerstone in consciousness: the ascending reticular activating system (ARAS), the thalamus and the posterior cingulate cortex. First, the upper brainstem is a main structure involved in arousal and awareness. As previously named by Moruzzi and Magoun (19), the ARAS is divided in four groups of nuclei: (1) the classical reticular nuclei (the nucleus cuneiforme, the deep mesencephalic nucleus, part of the pedonculo-pontine tegmental nucleus, and the pontis oralis nucleus), which send projections to the basal ganglia, the hypothalamus (20) and the intra-laminar thalamic nuclei (21), and then project to the cortex through the glutamate pathway; (2) the monoaminergic neurotransmitter system, which involves the locus coeruleus with norepinephrine (NE), the raphe nuclei with serotonin and the substantia nigra and ventral tegmental area with dopamine. This system directly targets the whole forebrain [cortex and subcortex—(22)]; (3) the cholinergic nuclei which include pedunculopontine and laterodorsal tegmental nuclei and project toward several thalamic nuclei and to the basal forebrain; and (4) the autonomic nuclei (parabrachial nucleus and periaqueductal gray matter) which targets the intra-laminar thalamic nuclei, the basal forebrain and other brainstem nuclei (23). Altogether, the ARAS has a main effect on wakefulness and vigilance (19, 24) and autonomic functions (25).

The thalamus is the second important structure involved in consciousness. It presents a complex architecture of nuclei organized as follows: from lateral to medial and from ventral to dorsal. Several specific thalamic relay nuclei communicate with the cortex according to their sensory and motor functions, and are a cornerstone of the contents of consciousness (23). Other thalamic nuclei project widely influence arousal and control the level of consciousness (23). Studies have shown that simultaneous bilateral thalamic infarction, as observed in the bilateral paramedian thalamic artery infarction or in the occlusion of the artery of Percheron, can induce a transient loss of consciousness at the onset of a stroke (26, 27). This temporary loss of consciousness shows that the thalamus is likely one of the primary sources for the ascending control of arousal.

Finally, the posterior cingulate cortex (PCC) is located in the medial part of the inferior parietal lobe and lies within the posteromedial cortex, which also includes the precuneal and retrosplenial cortices (28). This group of structures has been reported as the most metabolically active measured with fluorodeoxyglucose (FDG) PET-scan (29) during resting state (i.e., not performing any task) in healthy persons. The metabolic activity of these structures, using FDG-PET-scan, has also been associated to the level of consciousness in patients with DoC (30).

### Key Brain Networks

In addition to the specific structures that have been shown to be critical to consciousness, several brain networks have also been identified as equally important for consciousness recovery, and as a determining factor in the understanding of taVNS mechanisms of action. These networks are the default mode network (DMN), the external fronto-parietal network (ExN) and the salience network (SN). In order to demonstrate the association between brain structures outside these networks, which may also be relevant in the understanding of taVNS mechanisms of action, we also describe the Mesocircuit model (8), a theoretical model that exposes how anatomical structures influence each other, and how they may affect consciousness recovery [for a review see (31, 32)].

#### Default Mode Network, the Intrinsic Network

Raichle et al. (33) developed a hypothesis of a default mode in the brain. These authors noticed an important task-dependent decrease in activity in precise brain areas of healthy subjects; namely the medial prefrontal cortex (mPFC) bilaterally, the PCC and precuneal cortex. Later, they also included in the DMN the retrosplenial cortex, the inferior parietal lobule, the anterior cingulate cortex (ACC) and other brain regions, including the temporal lobe and part of the hippocampal formation (34). These structures were also highlighted in fMRI and diffusion spectrum imaging studies and described as critical hubs of the DMN (35, 36).

The DMN seems to be mainly active when people experience a resting state. It has been referred to as self-awareness (37) but also as the intrinsic network (38) because of its association to mental events like inner speech (39), day-dreaming or mind-wandering (40).

These correlations have encouraged the study of the DMN in DoC patients. Loss of connectivity within the DMN and with the thalamus (41) as well as altered patterns within the DMN when measured with FDG-PET-scan (42) have been reported in UWS patients. These observations have been confirmed using diffusion tensor imaging (43) and could be used as an indicator of consciousness state at the group level [coma < UWS < MCS < controls—(44, 45)].

#### The External Fronto-Parietal Network

While the DMN is associated to internal awareness (38), the lateral fronto-parietal network has been identified as the external network because it seems to be responsible for the relationship with the environment. The ExN, also called the task-positive network, is intimately linked to attention, response and action selection (46).

The ExN is characterized by the connectivity between the bilateral superior parietal and the lateral frontal cortices, while ipsilateral cortices are connected to each other by the ipsilateral superior parietal fasciculus (31). Its principal role is to draw attention to environmental stimuli in order to become aware of their presence (46), making it work closely with sensory subnetworks (38, 47–52). Due to the connections within these sensory subsystems, the ExN has been associated to external awareness [i.e., awareness of our environment—(53)].

Due to its relationship with the awareness of the environment, the ExN is likely to play an important role in the recovery of signs of consciousness in DoC patients. A (partially) preserved metabolism in this network, as measured with FDG-PET-scan, has been positively correlated with the CRS-R scores (42).

#### DMN and ExN Negative Connectivity

As defined by Fox et al. (54), the activity of the ExN regrouping regions routinely activated during goal-directed task performance, has been associated with a synchronized decrease in the DMN activation (54, 55). In that framework, Boly et al. (49) demonstrated that it is possible to predict a somatosensory stimulus perception according to the recorded brain activity 3 s before the stimulus presentation. More precisely, if the ExN activity was high, the somatosensory stimulus was perceived. However, if the DMN was the most active network, then the stimulus was not perceived, showing the likely negative correlation between both networks (49). This study confirmed the hypothesis supported by Raichle et al.: decreased PCC, precuneus and mPFC activity could be a way for the brain to reduce the resources devoted for general or intrinsic information (33). In other words, internal and external networks seem to be part of a competing system that opposes extrinsic to intrinsic information availability.

As negative connectivity between ExN and DMN seems to be a hallmark of a healthy state of consciousness, the lack of such a pattern is thought to play a role in DoC patients. Boly et al. (41) reported a significantly reduced internal-external networks negative connectivity in a single UWS patient compared to controls (41). Erratic deactivation patterns (56) as well as hyperconnectivity between DMN and ExN (45) have been reported in UWS patients. MCS patients, on the other hand, tend to have weak deactivation (56).

Therefore, recovering consciousness does not only require that the connectivity between the regions of ExN and DMN be preserved, but also that these two networks work in a particular synchronization, or rather asynchronously, in order to lead to self and environmental awareness.

#### Salience Network

The SN, a limbic-paralimbic network, represents another large-scale network. The fronto-insular cortex comprising the ventro-lateral prefrontal cortex and the anterior insula as well as the dorsal ACC form its core (57). The SN is involved in cognitive and affective tasks as it may drive attention and working memory resources to detect events and influence behavior (58, 59). In other words, it helps the brain to prioritize its next action to maintain homeostasis (60). Dynamic fMRI analyses showed that the SN has high temporal flexibility and spatiotemporal diversity which makes it uniquely positioned to facilitate interactions with multiple functional systems (59). Task-based functional neuroimaging studies have also identified a prominent role for the SN in switching between functional systems, i.e., between the ExN and the DMN (61, 62). Scientists have identified a particular cell class named von Economo neurons which seems to be present only in the SN (61) and have suggested that they are responsible for the network switching process as they provide a rapid relay to other parts of the brain (63).

A traumatic brain injury study by Bonnelle et al. (64) highlighted the need of SN structural integrity to efficiently regulate DMN activity. SN dysfunction has been reported to lead to a failure in DMN deactivation during focused task performance. Qin et al. (65) measured the SN's functional connectivity strength in DoC patients and in conscious patients with brain lesions. They reported a reduced connectivity in UWS patients compared to MCS and conscious patients when taken as a group. In addition, they showed significantly higher connectivity in MCS patients compared to UWS patients when performing analyses at the individual level.

### Mesocircuit

The Mesocircuit model (8) has been proposed to explain long-term regain of consciousness after severe traumatic brain injuries. This circuit is based on the main role of the central thalamus (the intralaminar nuclei and related paralaminar nuclei) and its connections to the striatum and the frontal cortex (See the Mesocircuit representation in the Supplementary Material). According to this hypothesis, the loss of central thalamus excitatory state, and consequently its excitatory cortical projections, is the cause of DoC. This state is the result of striatum dysfunction. Because striatum's neurons are particularly vulnerable to anoxia (66), it becomes unable to inhibit the globus pallidus internus which likely leads to the inhibition of the thalamus and pedonculopontine nuclei. Malfunction of the circuit reinforces the no-excitatory state of the thalamus. Evaluation of glucose metabolism revealed significantly higher metabolism in the globus pallidus and lower metabolism in the central thalamus in patients with a brain injury when compared to healthy subjects (67). Also, a significantly higher fractional anisotropy has been reported in the left hemisphere between the striatum and the globus pallidus of DoC patients when compared to healthy controls (68). Furthermore, the Mesocircuit model is able to integrate treatment mechanisms to explain their potential positive effects on DoC patients' clinical improvement (14, 69).

Altogether, consciousness requires: (1) active key brain structures; (2) within DMN and ExN connectivity; (3) a negative connectivity between the DMN and the ExN which is likely controlled by the SN; and (4) an intact cortico-striatal-thalamic-cortical loop, as described in the Mesocircuit model. In other words, to regain consciousness, these components have to be at least partially re-established, and they should be targeted by specific treatments to enhance patients' recovery.

## Vagus Nerve

### Anatomy and Functions

The vagus nerves are the tenth cranial nerve pair and are the principal nerves of the parasympathetic system. They are connected to four nuclei according to their different functions [for more details see (70)]. Afferences (80% of the vagal nerve sensory fibers) come from two main sources. The first type of afferent fibers carry general visceral information input from different sources such as lower pharynx, larynx, trachea, esophagus, and thoracic and abdominal viscera (including stretch and chemoreceptors). All visceral afferent fibers converge to the esophageal plexus and travel up in the right and left vagus nerves. They are joined by upper visceral afferences and together form the inferior vagal ganglion. They then enter the medulla and descend into the tractus solitarius to enter the caudal part of the nucleus of the tractus solitarius (71). From the nucleus, important connections are made with the spinal trigeminal nucleus and the reticular formation, including the locus coeruleus, the thalamus and the hypothalamus (72). The second type of afference comes from general somatic input, such as posterior meninges, conchae, skin on the back of the ear and in the external acoustic meatus and part of the tympanic membrane. Cadaver dissection made it possible to more precisely determine the vagal nerve branch anatomy on the ear and to observe that the cymba conchae was the only part consistently innervated by the auricular branch of the vagus nerve (73, 74). The auricular branch passes through the jugular foramen, enters the medulla and then ascends into the spinal trigeminal nucleus (71). The second-order axons project to the thalamus into two different nuclei; the first then projects toward the somatosensory cortex and the second projects toward the cingulate cortex (75, 76).

Efferences (20% of the vagal nerve sensory fibers) are also divided in two categories. The first group refers to special visceral efferent fibers that control swallowing and phonation. These fibers initiate from premotor, motor and other cortical areas, descend through the internal capsule and synapse onto motor neurons in the nucleus ambiguus in the medulla. They supply the striae muscles such as pharyngeal plexus, the superior, middle and inferior constrictors, levator palati, salpingopharyngeus, palatopharyngeus, palatoglossus, and the intrinsic muscles of the larynx. Second, the general visceral efferent fibers innervate smooth muscles and stimulate glands in the pharynx, larynx, thoracic and abdominal viscera, cardiac muscle, and the aortic bodies. These parasympathetic nerve cell bodies are located in the dorsal motor nucleus of the vagus nerve and in the medial side of the nucleus ambiguus (77).

## Unique Case of an Implanted Vagal Nerve Stimulation in a Patient With DoC

Implanted VNS has been investigated in a case-report of a UWS patient for which a vagal nerve stimulator was surgically implanted 15 years after a traumatic brain injury (15). The device was switched on 1 month after surgery at a starting intensity of 0.25 mA and increased by 0.25 mA each week until it reached 1.5 mA (pulse frequency: 30 Hz, pulse duration: 500 ms, stimulation cycle: intervals of 30 s of stimulation followed by 5 min of rest all day long). After 4 weeks of stimulation, when the intensity reached 1 mA, the authors noticed a clinical improvement, i.e., reproducible and consistent progress in general arousal, sustained attention, body motility and visual pursuit (CRS-R improved from 5 to 10). Three months after activation, a FDG-PET-scan study showed extensive increased activity in the occipito-parieto-frontal cortices, the basal ganglia and the thalamus regions. Between baseline and 6 month post-VNS, EEG results showed a significant increase in theta band power, which has been linked to consciousness (78). In addition, theta network centrality correlated with tDCS response and differentiated tDCS responders and non-responders (42). This increase in theta power was distributed over the occipito-parietal, inferior temporal and fronto-central regions, as well as at a deeper level, most likely localized in the insula. The recovery (i.e., increase in CRS-R scores) was linked to a rise in thalamo-cortical and fronto-parietal connectivity through a “bottom-up” fashion.

## Transcutaneous Auricular Vagal Nerve Stimulation

With the aim of better understanding the effects of taVNS on brain activity, studies that measured the effect of taVNS on the brain using resting state fMRI were selected from two different databases (Pubmed and Embase) and references were cross-checked. The next two sections describe the studies performed in healthy controls and in DoC patients respectively.

### Studies in Healthy Controls

Seven studies on healthy subjects have been selected (79–86). Stimulation parameters and methodological approaches were very heterogeneous and are summarized in Table 1. All studies reported brain activation or deactivation compared to sham stimulation (or to baseline).

**Table 1:** Healthy subject's fMRI activation/deactivation results after taVNS compared to sham (or to baseline) following anatomical order; brainstem, subcortical areas, and cortical areas.

**Studies**	**Kraus et al. (79)**	**Dietrich et al. (81)**	**Kraus et al. (80)**	**Frangos et al. (82)**	**Yakunina et al. (83)**	**Badran et al. (84)**	**Sclocco et al. (85)**	**Sclocco et al. (86)**
Objective of the study	Feasibility	Feasibility	Compare stim sites	Compare to sham	Compare stim sites	Compare to sham	Brainstem evaluation	Compare frequencies
Number of subjects	8	4	8/8	12	37	17	16	30
Age (mean ± SD) years	Range 20–37	30.0 ± 2.7	Range 20–37	32.6 ± 13.8	30.9 ± 8.2	25.8 ± 7.6	27.0 ± 6.6	29.0 ± 9.8
Gender (ratio F:M)	6:2	0:4	U	9:3	19:18	8:9	9:7	17:13
Design	Parallel	Crossover	Parallel	Crossover	Crossover	Crossover	Crossover	Crossover
Blinding	Single	None	Single	Single	Single	Single	Single	Single
Devices	TENS EMP2	Custom	TENS EMP2	NEMOS®	Custom	Custom	Custom	Custom
Stimulation site	Inner tragus L	Inner tragus L	Inner tragus L	Cymba conchae L	Cymba conchae L	Tragus L	Cymba conchae L	Cymba conchae L
**Comparative**								
Sham location	–	–	Lobe L	Lobe L	Lobe L	Lobe L	Lobe L	Cymba conchae L (no current)
Baseline	No stim	Resting-state		Resting-state	Resting-state	Resting-state	–	Resting-state
Frequency (Hz)	8	25	8	25	25	25	25	2, 10, 25, 100
Pulse width (μs)	20	250	20	250	500	500	450	300
Intensity (mA)	Low: 4 ± 1.0 High: 5 ± 1.0	4.0–8.0	4.0–5.0 under pain threshold	0.3–0.8	0.2–1.8 0.1 under pain threshold	0.1–5.1 200% sensation threshold	1.6 ± 2.4 4–5 on a 0–10 pain scale	Vary with fq 2: 7.2 ± 1.0 10: 6.5 ± 1.3 25: 5.9 ± 1.2 100: 5.6 ± 1.2
Duration	2 min off – 30 s on−60 s off X 4	100 s off - 50 s on X 4 – 100 s off	2 min off – 30 s on−1 min off X 4	2 min off – 7 min on−11 min off	30 s on−60 s off X 4	30 s off – 60 s on−60 s off X 2 – 60 s on−30 s off	Match the breathing cycle for a total of 8 min	Match the breathing cycle for a total of 8.5 min
fMRI technique	1.5 T	1.5 T	1.5 T	3.0 T 12-Channel head coil	3.0 T 32-Channel head coil	3.0 T 32-Channel head coil	7.0 T 32-Channel and birdcage transmit coil	3.0 T 64-Channel head/neck coil
**Brainstem areas**								
Spinal trigeminal nucleus	–	–	–	**↑** bilat	–	nd*	**↑** ipsi	**↑** ipsi
Nucleus of the tractus solitarius	–	–	**↓**	**↑** ipsi	**↑** bilat	nd*	**↑** ipsi	**↑** ipsi
Locus coeruleus	–	**↑** ipsi	**↓**	**↑** bilat	**↑** bilat	nd*	**↑** cont	**↑** cont (2 Hz) **↑** bilat (100 Hz)
Raphe nuclei	–	–	–	**↑** bilat	–	nd*	**↑** cont	**↑** bilat (2,100)
**Subcortical areas**								
Thalamus	(↑c)	**↑** ipsi > cont	nd	**↑** bilat	**↑** bilat	nd	–	–
Caudate	(nd)	nd	–	?(↑c)	**↑** bilat	**↑** cont	–	–
Nucleus accumbens	(nd)	**↓** cont	–	**↑** cont (↑b)	–	nd	–	–
Putamen	(nd)	nd	–	?(↑c)	nd (nd)	nd	–	–
Amygdala	(↓ b)	nd	–	**↑** cont	nd (↓b)	nd	–	–
Hippocam-pus	(↓ i)	nd	–	**↓** bilat	nd (↓b)	nd	–	–
Parahippocampus	(↓ b)	nd	**↓** ipsi (↓i)	**↓** bilat	nd (↓b)	nd	–	–
Insula	(↑ b)	**↑** ipsi	**↑** ipsi (↑i)	**↑** bilat	nd (↓i)	nd (↑b)	–	–
Hypotha-lamus	(nd)	nd	–	**↓** bilat	nd (nd)	nd	–	–
Cerebellum	(nd)	**↓** cont	–	**↑** bilat	**↑** bilat	**↑** bilat (↑i)	–	–
**Cortical areas**								
mPFC	(↓b)	**↑** ipsi	**↑** bilat (↑b)	–	nd (↓b)	**↑** ipsi	–	–
Orbitofron-tal cortex	(nd)	nd	–	**↑** cont	–	nd	–	–
Lateral Frontal cortex	(↑i)	–	–	–	nd (↓c)	nd (↑b)	–	–
ACC	(↑c)	nd	nd (↑c)	**↑** bilat	nd (↓b)	**↑** cont	–	–
Pre-central gyrus	(↑b)	nd	–	–	nd (↓b)	nd	–	–
Post-central gyrus		**↑** bilat	–	**↑** bilat	nd (↓b)	nd (↑c)	–	–
Paracentral lobule	(↓b)		–	**↑** bilat	–	nd	–	–
PCC/precuneus	(↓b)	**↑** ipsi	nd (↓i)	**↑** bilat	nd (↓b)	nd	–	–
Parietal cortex	(nd)	nd	nd (↑c)	–	nd (nd)	nd	–	–
Temporal middle	(↓b)	–	–	–	nd (↓b)	nd	–	–
Occipital lobe	(nd)	nd	–	–	nd (↓i>c)	nd	–	–

ACC, anterior cingulate cortex; Bilat/b, bilateral; Cont/c, contralateral to the stimulated ear; fq, frequency; Ipsi/i, ipsilateral to the stimulated ear; L, left; mPFC, medial prefrontal cortex; min, minutes; nd, not detected; PCC, posterior cingulate cortex; s, seconds; stim, stimulation; ?, unknown; ↑, activation; ↓, deactivation; (), results between parentheses are taVNS vs baseline; results out of parentheses are taVNS vs sham; –, not analyzed; *, lack of results in the brainstem results may be biased due to the methodological approach used (not accurate for the brainstem).

First, two feasibility studies looked at the taVNS effects on the brain with small cohorts of participants (79, 81). Kraus et al. (79) used the Adjective Mood Scale score and compared the stimulation of the inner tragus to a controlled condition (i.e., no stimulation). The authors showed an improved average score, suggesting a significantly improved mood following stimulation. Then, they compared low and high intensity stimulations (low: 4 ± 1.0 mA, high: 5 ± 1.0 mA) using fMRI. Activity modulations were detected in a greater number of structures after high-intensity stimulation compared to low-intensity stimulation. Common modulated structure analyses led to higher BOLD signal in the left hemisphere areas at high-intensity stimulations compared to low-intensity stimulations, and to higher BOLD signals in the right hemisphere areas at low-intensity taVNS compared to high-intensity taVNS. Lastly, they compared the ear lobe to the controlled condition and showed ipsilateral activation of temporal gyrus, PCC, contralateral activation of the thalamus, insular cortex and cingulate gyrus, and a deactivation of the bilateral paracentral lobule and contralateral parahippocampal gyrus. In another study, Dietrich et al. (81) reported activation and deactivation patterns after inner tragus stimulations when compared to the baseline, but they did not use a sham condition. They also measured effects on blood pressure, heart rate and laser Doppler flow without noticing changes in these parameters (81).

Three placebo controlled studies compared different stimulation sites to the ear lobe (80, 82, 83). First, Kraus et al. (80) designed a study to identify the best site to stimulate the vagus nerve in the ear and compared stimulation effects of the anterior or the posterior wall of the auditory canal. They measured deactivation of the nucleus of the tractus solitarius and the locus coeruleus after stimulation of the anterior wall. No difference was shown when the stimulation was at the posterior wall or at the lobe for these two structures. Then, Frangos et al. (82) performed stimulation at low-intensity (0.3 to 0.8 mA) at the cymba conchae to modulate electrical activity of the brainstem and the brain. The authors also reported that the greatest effect of the stimulation was measured during the post-stimulation period lasting up to 11 min. Lastly, they showed an increase in the activation of the nucleus of the tractus solitarius, not only in the caudal part. The activation seemed to extend superiorly through the medulla oblongata. The other study compared stimulation localization sites: inner tragus, cymba conchae, infero-posterior wall of the ear with the ear lobe (83). The fMRI results revealed higher signals in the nucleus of the tractus solitarius and the locus coeruleus and thus confirmed that the first two stimulation sites were associated to the vagus nerve. This activation pattern was not demonstrated after the stimulation of the infero-posterior wall neither at the ear lobe (87).

One more study compared the tragus stimulation to the ear lobe during two different sessions separated by at least 24 h (84). They reported a significant difference in the activation pattern produced by the inner tragus stimulation when compared to the ear lobe after only 1 min of stimulation.

Finally, another research group focused on taVNS' brainstem effects (85, 86). The authors matched stimulation with the respiratory cycle and stimulated patients during the inhalation or the exhalation phase on two different fMRI runs. A significant difference between both phases was reported, with the exhalation phase leading to a higher activation pattern in the nucleus of the tractus solitarius, ambiguus, and olivary nuclei, as well as in the locus coeruleus and the raphe nuclei. Then, they investigated the effects of stimulation during the exhalation phase at different frequencies (2, 10, 25, and 100 Hz) and recorded brainstem fMRI signal for each of them. Stimulation delivered at 100 Hz resulted in a significant increase in the fMRI signal in all targeted brainstem nuclei (ipsilateral periaqueductal and ambiguous nuclei in addition to the nuclei described in the Table 1) compared to no stimulation. Significant differences were also reported at 2 Hz, but only in the dorsal raphe and the contralateral locus coeruleus (86).

### Studies in Patients With DoC

One case-report investigated taVNS on a 73 year-old woman in UWS (50 days post-cardiopulmonary resuscitation) (16). Prior to taVNS, the patient was able to open her eyes without stimulation and had a clear sleep-wake cycle. Her CRS-R total score was 6 (out of 23) and stable over several weeks. The patient then received 4 weeks of bilateral stimulation which led to an improvement in the CRS-R score (from 6 to 13 in motor and oromotor subscales) and a change in diagnosis (from UWS to MCS). After 4 weeks of treatment, the authors reported that the thalamus and PCC/precuneus were activated by taVNS as measured by fMRI. They also showed that taVNS increased the functional connectivity between the PCC/precuneus (used as seed) and the thalamus, medial-ventral PFC, hypothalamus and superior temporal gyrus, with a decreased functional connectivity between the PCC/precuneus and the cerebellum.

A case study series also reported behavioral improvements after taVNS stimulation (17) in 5 out of 14 patients who were stimulated at the left tragus for 4 weeks. The six UWS and eight MCS patients were in a prolonged state (more than 6 months), and had at least five consecutive stable CRS-R scores. The CRS-R was administered at baseline during each week of stimulation and 4 weeks after the end of stimulation. The authors observed that the CRS-R score improved for one MCS patient after 4 weeks of treatment. Furthermore, 4 weeks after the end of stimulation, four additional MCS patients recovered at least one sign of consciousness and were considered as responders. At this time-point, the first patient who showed improvement maintained his/her CRS-R score. Four of the responders showed improvement in only one CRS-R subscale (motor subscale for three of them; visual subscale for one of them) and one patient improved in more than one subscale (unspecified). No neuroimaging nor electrophysiological assessment was performed to assess the mechanisms associated to taVNS and improvements of consciousness.

Behavioral improvement was also reported in a study of five traumatic brain injury participants (18). After 8 weeks of 4 h of daily stimulation, four out of five participants improved their CRS-R total scores and three of them presented a better diagnosis, with two of them becoming EMCS. The majority of participants achieved the full 4 h of daily stimulation (median: 43 days out of 56, range: 28–52). The main reported reasons to explain the lack of stimulation were in case of medical complications not related to the taVNS or loss of skin contact. The Table 2 summarized sample characteristics and findings.

**Table 2:** Summary of taVNS studies in DoC patients.

**Studies**	**Yu et al. (16)**	**Noé et al. (17)**	**Hakon et al. (18)**
Subjects	1 UWS	6 UWS 8 MCS	3 UWS 2 MCS
Age (mean ± SD)	73	40.2 ± 16.1	Median: 67 Range: 21–80
Etiology	Anoxic: 1	Traumatic: 7 Anoxic: 4 Vascular: 3	Traumatic: 5
Time since injury (mean ± SD)	50 days	12.1 ± 6.4 months	Median: 41 days Range: 31–95 days
Stim site	Cymba conchae bilat	Left tragus	Cymba conchae
Sham	–	–	–
Device	Custom	Parasym®CE	NEMOS®
Frequency (Hz)	20	20	25
Pulse width (μs)	<1,000	250	250
Intensity (mA)	4.0–6.0	1.5	0.5 the first 3 days and 1 afterwards
Duration	30 min twice a day for 4 weeks	30 min twice a day, 5 days/week for 8 weeks	4 h daily interval of 30 s on and 30 s off for 8 weeks
fMRI findings (3.0T)	Thalamus ↑ PCC/ precuneus ↑	–	–
Behavioral findings	Baseline CRS-R: 6 CRS-R at 4 weeks: 13 Improvement in motor and oromotor subscales Diagnosis: UWS → MCS	Responders (showed at least one new sign of consciousness) 0/6 UWS 5/8 MCS Improvement in - motor subscale: 3 patients; - visual subscale: 1 patient - >1 subscale: 1 patient	Diagnosis/ CRS-R score UWS → EMCS/ 5 → 22 UWS → MCS/ 3 → 6 UWS → UWS/ 2 → 3 MCS → MCS/ 12 → 12 MCS → EMCS/ 10 → 23

*CRS-R, coma recovery scale-revised; EMCS, emergence of minimal conscious state MCS, minimal conscious state; UWS, unresponsive wakefulness syndrome; fMRI, functional magnetic resonance imaging; Hz, Hertz; mA, milliamperes; μs, microseconds; s, seconds; SD, standard deviation; T, Tesla*.

## Model's Proposal

Based on the state-of-the-art presented above, we propose a model to explain how taVNS influences brain activity that can be applied to DoC patients and explain the process of consciousness recovery.

### The Vagal Cortical Pathways Model

**Figure 1:**
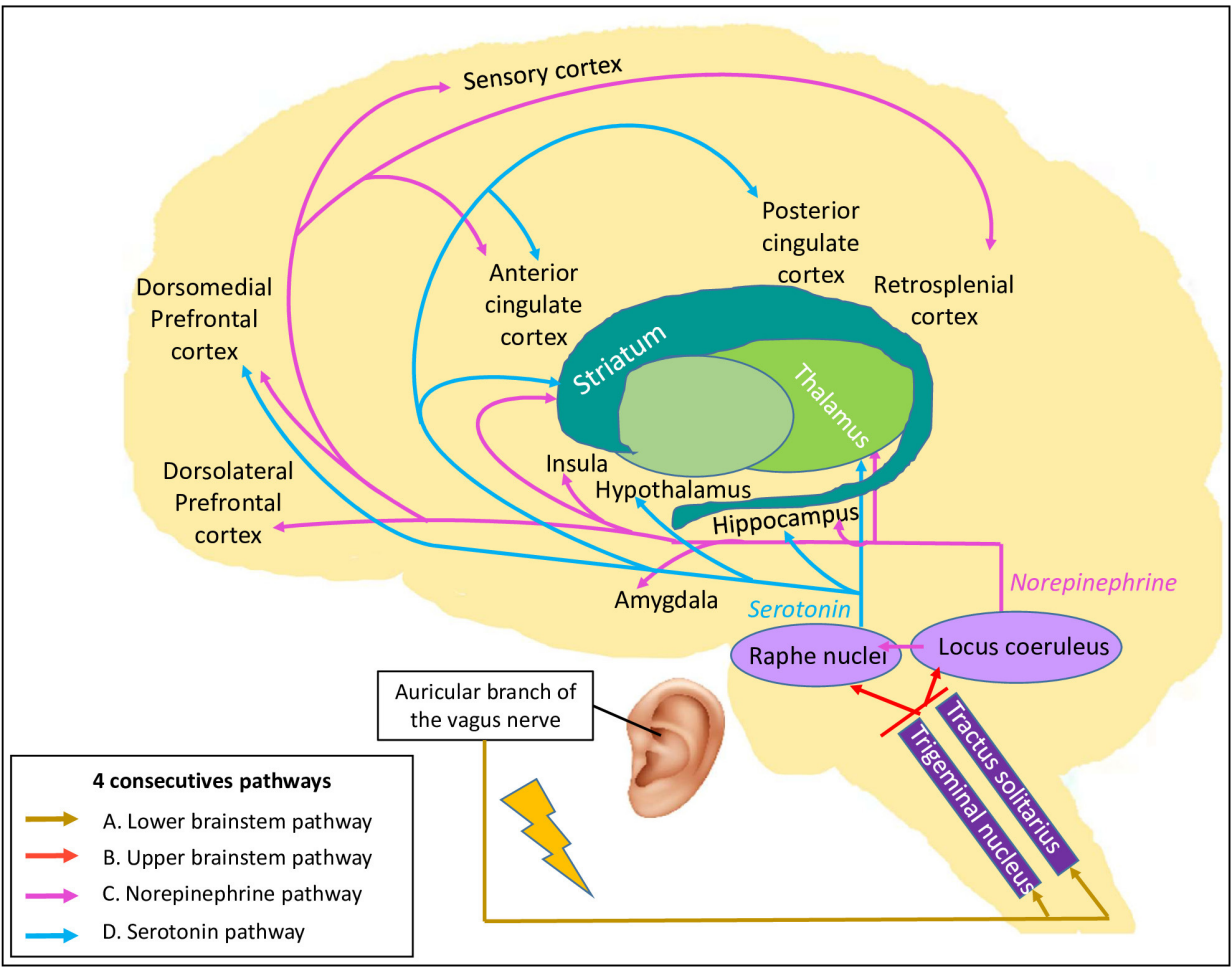
The Vagal Cortical Pathways model: overall four consecutive pathways of the transcutaneous auricular vagal nerve stimulation. By stimulating the auricular branch of the vagus nerve, it leads to **(A)** the activation of the trigeminal nucleus and tractus of the solitarius nucleus located in the lower brainstem (gold arrows). Their activation leads to **(B)** the activation of the locus coeruleus and the raphe nuclei localized in the upper brainstem (red arrows). Then, **(C)** the locus coeruleus produces norepinephrine and modulates global brain activity (pink arrows). Finally, **(D)** the raphe nuclei produce serotonin, which also targets the brain, especially some structures of the limbic system and the frontal cortex (blue arrows).

Based on the vagus nerve anatomy, we proposed four consecutive pathways to explain how taVNS may influence brain activity, represented in the Vagal Cortical Pathways model (Figure 1). First, we reasoned that taVNS leads to the activation of the spinal trigeminal nucleus which, in turn, leads to the activation of the tractus of the solitarius nucleus (88), both nuclei located in the lower brainstem (pathway A of the vagal cortical model).

Second, these nuclei project to the upper brainstem, directly to the locus coeruleus (89) and indirectly via the nucleus paragigantocellularis (90), and to the raphe nuclei (91) (pathway B of the vagal cortical model).

Third, activation of the locus coeruleus results in the release of NE (92, 93) which, in turn, has widespread effects on many brain areas (94) (pathway C of the vagal cortical model). NE likely acts at different levels (synaptic, cellular, microcircuit and network) and modulates sensory-motor responses, as well as prefrontal activity (95). All levels of interaction facilitate cognitive functions, such as attention, emotion, decision-making, motivation, learning and memory (90, 96–99). The rise in NE availability leads to large-scale brain network reconfiguration in the areas densely innervated by the locus coeruleus (99). A first cluster regroups the insular area, the ACC, the ventro-medial striatum, the nucleus accumbens, the globus pallidus, the thalamus, and the hippocampus, while a second cluster includes the amygdala, the claustrum, the sub-thalamic nucleus, and the zona incerta (99, 100). These networks are involved in stress reaction (100) and should facilitate task performance (101).

Last, raphe nuclei activation causes serotonin release and affects specific brain areas (pathway D of the vagal cortical model). This neurotransmitter has an affinity for selective brain areas, such as the hippocampus, hypothalamus, thalamus, nucleus accumbens, cerebellum, anterior and posterior cingulate cortices and dorsomedial prefrontal cortex (102). These projections increase the activity in the DMN and decrease the activity in the sensory-motor network (103, 104).

#### Hypothesis Challenge

To challenge the Vagal Cortical Pathways model, we looked at the taVNS fMRI studies mentioned in Table 1 and used their results to corroborate each of the four pathways of the proposed model.

The first pathway (pathway A) to challenge is the activation of lower brainstem nuclei. Spinal trigeminal nucleus and tractus of the solitarius nucleus have been reported in four and five fMRI studies, respectively. Three studies supported the activation of the spinal trigeminal nucleus (82, 85, 86) while one did not report any difference in this structure (84). Activation of the tractus of solitarius nucleus was identified in four studies (82, 83, 85, 86) whereas no change was measured by Badran et al. (84).

According to our model, lower brainstem activation should lead to the activation of the upper brainstem (pathway B), especially the ARAS, through the locus coeruleus and the raphe nuclei. As expected, locus coeruleus activation was reported in five out of the seven studies (81–83, 85, 86). Three studies showed an activation of the raphe nuclei following taVNS, either contralateral to the stimulation (82) or bilaterally (85, 86), while Badran et al. reported no difference (84). Altogether, the studies that analyzed the brainstem with an appropriate protocol (82, 83, 85, 86) supported the activation of the lower and upper brainstem, thereby confirming our hypotheses.

The NE (pathway C) and serotonin (pathway D) pathways share some targeted brain areas: the thalamus, the striatum, the hippocampus, the medial prefrontal cortex and the post-central gyrus (sensory cortex). Thalamic activation is important for both pathways. A higher activation of the thalamus following taVNS was reported in three out of four studies investigating this hub (81–83). The striatum, which regroups caudate, putamen and nucleus accumbens, were reported to be more active in at least one striatum part (82–84). Dietrich et al. (81) did not show any differences in the striatum, except for a deactivation in the contralateral nucleus accumbens (81). For the hippocampus, either no difference was reported (81, 83, 84) or no statement results were made (82). Two studies showed an activation of the ipsilateral medial prefrontal cortices (81, 84) while Yakunina et al. (83) showed no difference, and Frangos et al. (82) did not analyze these brain areas. All studies supported sensory cortex activity: two of them demonstrated no difference between sham and active stimulation (83, 84), while two others highlighted a bilateral activation (81, 82). In summary, most of the studies reported a higher activation in most of the shared structures, except for the hippocampus. Altogether, the majority of these results matches our hypothesis.

Specific brain areas associated to NE pathway (pathway C) include the amygdala, the insula, the ACC and the lateral prefrontal cortex. One out of four studies (82) mentioned a significantly higher activation of the amygdala, while others reported no difference when comparing taVNS to sham stimulation (81, 83, 84). Regarding insula, a higher activation was outlined in two studies (81, 82) but not in the other two (83, 84). The ACC was reported to be more active in two studies (82, 84) while no difference between sham and stimulation were shown in the other two studies (81, 83). Finally, the lateral prefrontal cortex was not influenced by taVNS (83, 84) or was not analyzed (81, 82). Whereas, the results varied from one study to another, with some of them supporting higher activation and others highlighting no difference, no study reported deactivation. However, when compared to baseline, results were not consistent. These findings were only partially aligned with the hypothesis of the NE pathway activation.

Last, the serotonin pathway (pathway D) projects to specific brain regions, such as the hypothalamus, the PCC and the cerebellum. One study detected a significant difference in the hypothalamus, as a bilateral deactivation, when compared to sham and baseline (82). Furthermore, all studies showed bilateral activation of the cerebellum except for Dietrich et al. (81) who reported a contralateral deactivation by taVNS. Overall, these results were inconclusive with respect to the activation of the serotonin pathway.

Due to methodological [stimulation site inconsistent with the auricular branch of the vagus nerve innervation site (87)] and data processing (uncorrected *p*-values in a cohort of eight participants, and the report of a greater activation of the nucleus of the tractus solitarius and locus coeruleus during the sham condition compared to active stimulation) inconsistencies in two studies (79, 80), we decided to exclude them from the hypothesis challenge. Questioning of the results (79) has already been raised (81, 87).

To summarize, most of the fMRI studies on healthy subjects seemed to support the activation of the lower and upper brainstem following taVNS (pathways A and B of the vagal cortical model). Although not unequivocal in all fMRI studies, the activation of neurotransmitter pathways by taVNS, especially the NE pathway (pathway C), is likely promoted (pathways C and D of the vagal cortical model).

### Vagal Cortical Pathways Model Applied to DoC

**Figure 2:**
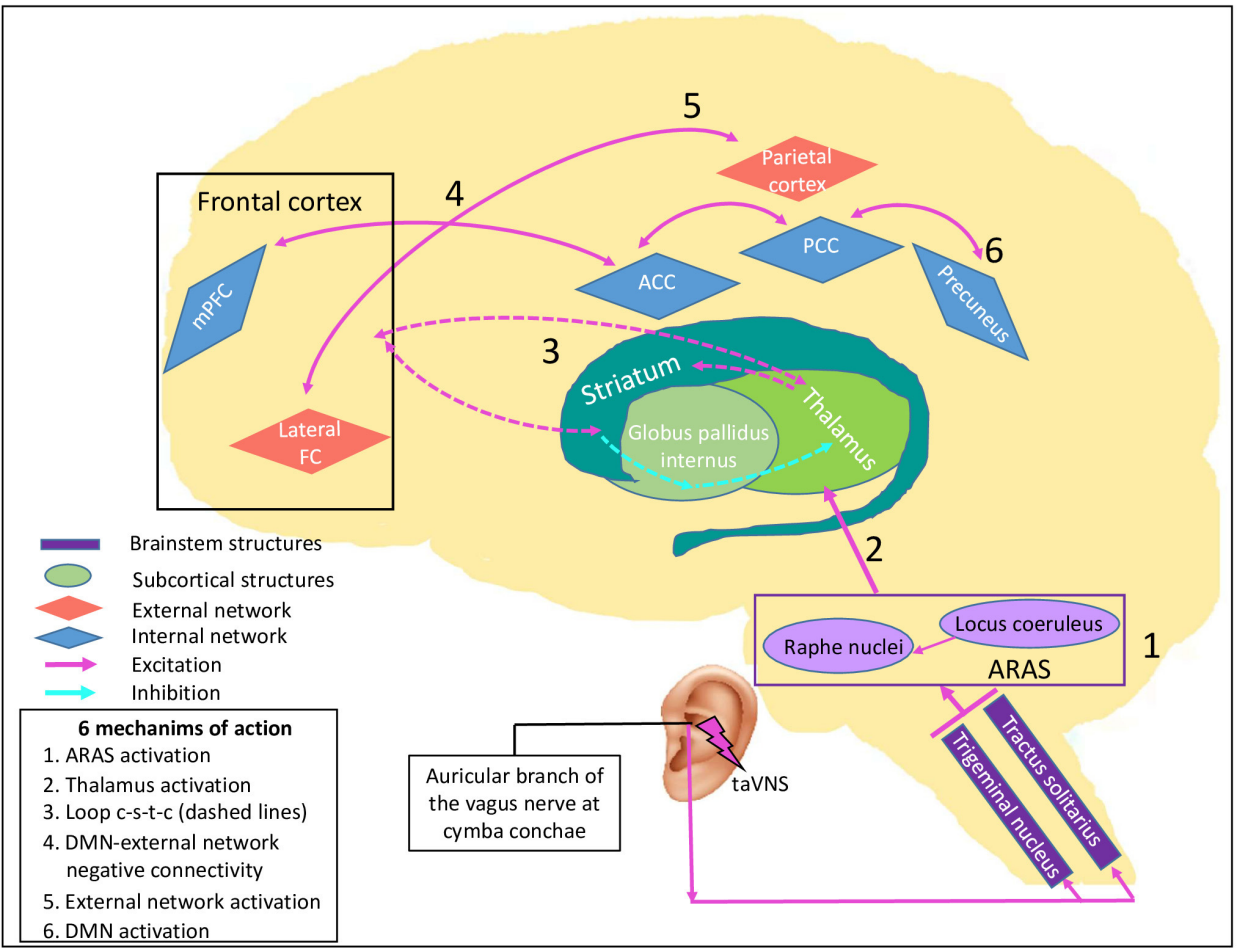
taVNS' specific potential effects on consciousness recovery processes. This figure illustrates the effects of proposed pathways of transcutaneous auricular vagal nerve stimulation on a damaged brain causing disorders of consciousness, leading to six mechanisms of action. By stimulating the auricular branch of the vagus nerve, taVNS activates the caudal part of the trigeminal nucleus and of the tractus solitarius in the lower brainstem. Their neurons synapse and activate the locus coeruleus and the raphe nuclei in the upper brainstem which synthetize NE and serotonin respectively. 1) Both nuclei are part of the ascending reticular activating system (ARAS, purple rectangle), have a direct effect on arousal, and 2) project directly to the thalamus. Through their excitatory neurotransmitters and direct projections, it leads to 3) re-establishment of the cortico-striatal-thalamic-cortical loop (dashed arrows), 4) promotion of negative connectivity between external network (red diamonds) and internal network (blue diamonds) because of a higher activity of the salience network, 5) increase in external network activity and strength connectivity through NE projections, and 6) increase in DMN activity through serotonin projections. Purple rectangles represent brainstem nuclei, green round shapes represent subcortical structures and diamonds represent cortical structures, in blue the intrinsic network and in red the external network. ACC: anterior cingulate cortex, ARAS: ascending reticular activating system, DMN: default mode network, lateral FC: lateral frontal cortex, loop c-s-t-c: cortico-striatal-thalamic-cortical loop, mPFC: medial prefrontal cortex, PCC: posterior cingulate cortex, taVNS: transcutaneous auricular vagal nerve stimulation.

Based on the neural correlates of consciousness already discussed, we think that it is possible to expand the suggested Vagal Cortical Pathways model to explain recovery in patients with DoC. We hypothesize that taVNS leads to an improved level of consciousness of DoC patients following the four pathways described above, and explained by six specific mechanisms of action on brain activity and consciousness level (Figure 2).

First, according to the pathways A and B, there should be an activation of the locus coeruleus and the raphe nuclei. These nuclei are located in the ARAS and should participate in the improvement of arousal and some autonomic functions (mechanism 1). Once the upper brainstem is activated, subcortical structures and neurotransmitter pathways should be activated through direct projections. Specifically, the ARAS projects directly to the thalamus and should favor its activation (mechanism 2). The thalamus is a key structure that has the potential to modulate arousal and consciousness and is part of both the NE and serotonin pathways (pathways C and D of the Vagal Cortical Pathways model).

The increase in thalamic activity suggests a consequent stimulation of the striatum, which is also a direct projection of the raphe nuclei, and consequently should help to re-establish the cortico-striatal-thalamic-cortical loop (mechanism 3). This should have a major impact on consciousness according to the Mesocircuit hypothesis (8) (Supplementary Material).

In addition, the NE pathway (pathway C) may influence consciousness in two ways. First, projections of this pathway promote activity in many structures of the SN (105, 106). Through activation, it facilitates the switch from DMN to ExN (mechanism 4) which should also contribute to improve attention (107). In other words, it could improve negative connectivity between these two networks (108). As mentioned, this dichotomy is correlated to higher levels of consciousness and its enhancement is part of consciousness recovery in DoC patients (42, 45).

Second, reports suggest that NE boost (pathway C) leads to a better capacity of reaction to stimuli and is correlated to an increase in gamma coherence (109). In DoC patients, the gamma rhythm has also been studied (110–112). Gamma coherence enhancement and higher connectivity within the ExN is demonstrated in MCS and normal subjects in response to the sensory stimuli, while no change is found in UWS patients (110). Consequently, we believe that taVNS has the potential to promote ExN activity (mechanism 5), which is essential to improve interaction with the environment. It could also be a possible explanation to the higher CRS-R scores measured in the DoC's case report and series involving taVNS (16–18).

Finally, the serotonin pathway (pathway D) could lead to an enhancement of connectivity within the DMN. By its projections to mPFC, ACC, and PCC, it has the potential to promote connectivity between these structures (mechanism 6). Higher DMN connectivity is correlated to a higher level of consciousness (44, 113).

#### Hypothesis Challenge

Proposed mechanisms of action in DoC patients are supported by fMRI studies mentioned above in Tables 1, 2. The ARAS activation through the locus coeruleus and the raphe nuclei (mechanism 1) has been demonstrated in the hypothesis challenge of the pathway B detailed above.

Bilateral thalamic activation (mechanism 2) was reported in most of the studies (16, 81–83). Badran et al. (84) reported no significant difference and Sclocco et al. (85, 86) did not investigate this structure. The taVNS studies that analyzed striatum activity in healthy subjects are reported above in section Hypothesis Challenge. Results concerning the thalamus (mechanism 2) and striatum activation (mechanism 3) are not totally consistent yet they suggest a thalamic activation (mechanism 2). Further investigations are needed before reaching any conclusion about the striatum (mechanism 3).

When we challenge changes in connectivity, conclusions are more difficult to draw because no study was designed for this purpose. However, activation of the insula was reported following taVNS (81, 82, 84), which could be suggestive of a higher activity in the SN and consequently promote the negative connectivity between ExN and DMN (mechanism 4).

It was not possible to confirm or disprove our hypothesis about connectivity within the ExN (mechanism 5) by analyzing the fMRI results. Indeed, fMRI data collections were acquired in a resting state, promoting DMN over ExN. Furthermore, ExN hubs have never been used as seeds to measure ExN connectivity in the selected studies. One could extrapolate that behavioral improvement is associated to an increase in ExN strength connectivity (mechanism 5) or in the negative connectivity with DMN (mechanism 4) or both. However, this was not directly measured.

Finally, higher connectivity within the DMN (mechanism 6) was reported in some studies (81, 82, 84). These studies showed activation of at least two brain areas among mPFC, ACC and PCC. This could be an indirect evidence of higher connectivity in the DMN core.

## Discussion

Based on existing anatomical and neurophysiological studies, we developed the Vagal Cortical Pathways model. We demonstrated that this model is supported by the majority of the findings reported in taVNS-based fMRI studies on healthy subjects. Moreover, we applied this model in the context of DoC, hypothesizing six mechanisms of action to explain the potential role of taVNS in the recovery of consciousness.

The selected studies did not confirm or disprove all our hypothesis on taVNS-induced activation pathways and how they could be applied to DoC patients. One important limitation of this article is the number of studies using taVNS and neuroimaging published in the literature. This analysis only includes fMRI studies because this technique provides one of the most accurate spatial information allowing comparisons between studies. Nevertheless, many methodological parameters including stimulation site, comparative features (sham vs. control), stimulation characteristics (duration, on-off pattern, pulse width, and frequency) and the quality of fMRI data analysis vary among studies. Consequently, the comparison between studies was restrained.

To confirm the Vagal Cortical Pathways model and the associated mechanisms of action, a double-blind crossover study that combines clinical and neuroimaging measures should be conducted. Cerebral activity measurements during task performance and resting state should be the method of choice. Moreover, fMRI studies should always include brainstem analysis to confirm the activation of the vagal nerve pathway (pathways A and B of the Vagal Cortical Pathways model). This should be used as a guarantee of quality in studies using taVNS because it proves without any doubt, that the vagal nerve is stimulated (87, 114). In addition, subcortical areas, such as the thalamus and the striatum should be systematically analyzed. To estimate brain connectivity, the thalamus, the frontal lateral cortex, the ACC or the insula and the PCC should be used as seed regions in fMRI analyses. Using this methodological approach, it should be possible to confirm or refute the four taVNS-associated pathways proposed in the Vagal Cortical Pathways model and its six specific mechanisms of action explaining the recovery processes of DoC patients.

The methodology may have a major influence on findings related to the application of taVNS. After implanting VNS in rats, Dorr and Debonnel (115) described different time periods needed to increase the NE and serotonin firing rates. They compared firing rates after 1 h as well as 1, 3, 14, 21, and 90 days of stimulation. After 1 h, the NE firing rate was significantly increased while 14 days were required to observe a rise in the serotonin firing rate. These differential firing rates are likely to be similar in humans. In this situation, short stimulations such as the ones applied in the healthy subject studies mentioned in this article, might induce the NE pathway activation (pathway C of the Vagal Cortical Pathways model), but not the serotonin pathway activation (pathway D of the Vagal Cortical Pathways model). A longer period of stimulation may be needed to promote the serotonin pathway activation. Of note, taVNS has been recommended for major depression disorders and some studies showed mood improvement after four weeks of regular use (116–119) which could be attributed to higher release of NE and serotonin.

Other questions regarding the possible mechanisms of taVNS remain unanswered. Are dopamine and gamma-aminobutyric acid (GABA) implied in taVNS mechanisms of action? In this case, how could they influence the pathways described? Some studies have suggested that NE and serotonin may modulate dopamine release (104). Furthermore, GABA_A_ has been hypothesized to be a NE modulator (120, 121). Dopamine and GABA_A_ agonists already showed positive effects on DoC patients (122, 123) and were potentially part of another mechanism of action involved in taVNS-induced consciousness recovery. However, to date, only indirect signs of GABA modulation by taVNS have been reported in healthy subjects (124–126). Additionally, brain injury could disrupt structure projections but could also have a more general impact on the brain through changes in the blood brain barrier (127) and inflammatory processes (128), also having an impact on the neurotransmitter release (129). It remains unknown how these brain dysfunctions, disconnected brain areas and acquired brain injuries affect taVNS pathways.

## Conclusion

The Vagal Cortical Pathways model describes four consecutive pathways explaining the influence of taVNS on brain activity: (A) direct activation of the lower brainstem nuclei; (B) activation of the locus coeruleus and the raphe nuclei in the upper brainstem; (C) NE projections with a general influence on the brain; and (D) serotonin projections to limbic and DMN structures. In the particular context of DoC patients, we went one step further and proposed six specific mechanisms of action of taVNS underlying consciousness recovery: (1) activation of the ARAS, (2) activation of the thalamus, (3) activation of the striatum and re-establishment of the cortico-striato-thalamo-cortical loop, (4) improvement in DMN and ExN negative connectivity through the NE pathway and the activation of the SN, (5) activity and connectivity improvements within the ExN through the NE pathway, and (6) connectivity enhancement within the DMN through the serotonin pathway. Considering these six components, we believe that taVNS represents a remarkable potential approach to modulate arousal and awareness in DoC patients. If the proposed Vagal Cortical Pathways model is confirmed by prospective controlled clinical trials, taVNS should be considered as a valuable bottom-up therapeutic approach for DoC patients.

## Author Contributions

M-MB: conceptualization, investigation, writing—original draft, visualization, and editing. AT: investigation, writing—review and editing, supervision, and project administration. OG, BS, and SL: writing—review and editing. All authors gave final approval of the manuscript.

## Conflict of Interest

The authors declare that the research was conducted in the absence of any commercial or financial relationships that could be construed as a potential conflict of interest. The reviewer CD declared a past co-authorship with one of the authors SL to the handling editor.

## Abbreviations

ACC: anterior cingulate cortex
ARAS: ascending reticular activating system
CRS-R: coma recovery scale-revised
DBS: deep brain stimulation
DMN: default mode network
DoC: disorder of consciousness
EEG: electroencephalography
EMCS: emergence from minimally conscious state
ExN: external fronto-parietal network
FDG: fluorodeoxyglucose
fMRI: functional magnetic resonance imaging
GABA_A_: γ*-*aminobutyric acid type A
mA: milliamperes
MCS: minimally conscious state
MDD: major depressive disorder
mPFC: medial prefrontal cortex
NE: norepinephrine
NMDA: N-méthyl-D-aspartate
PCC: posterior cingulate cortex
PET: positron emission tomography
rTMS: repetitive transcranial magnetic stimulation
SN: salience network
taVNS: transcutaneous auricular vagal nerve stimulation
tDCS: transcranial direct current stimulation
UWS: unresponsive wakefulness syndrome
VNS: vagal nerve stimulation.
