# Supplementary figures and images for: Transcutaneous Auricular Vagal Nerve Stimulation and Disorders of Consciousness: A Hypothesis for Mechanisms of Action
Source: Front Neurol. 2020 Aug 25;11:933. doi: 10.3389/fneur.2020.00933 (PMC7477388; doi:10.3389/fneur.2020.00933)

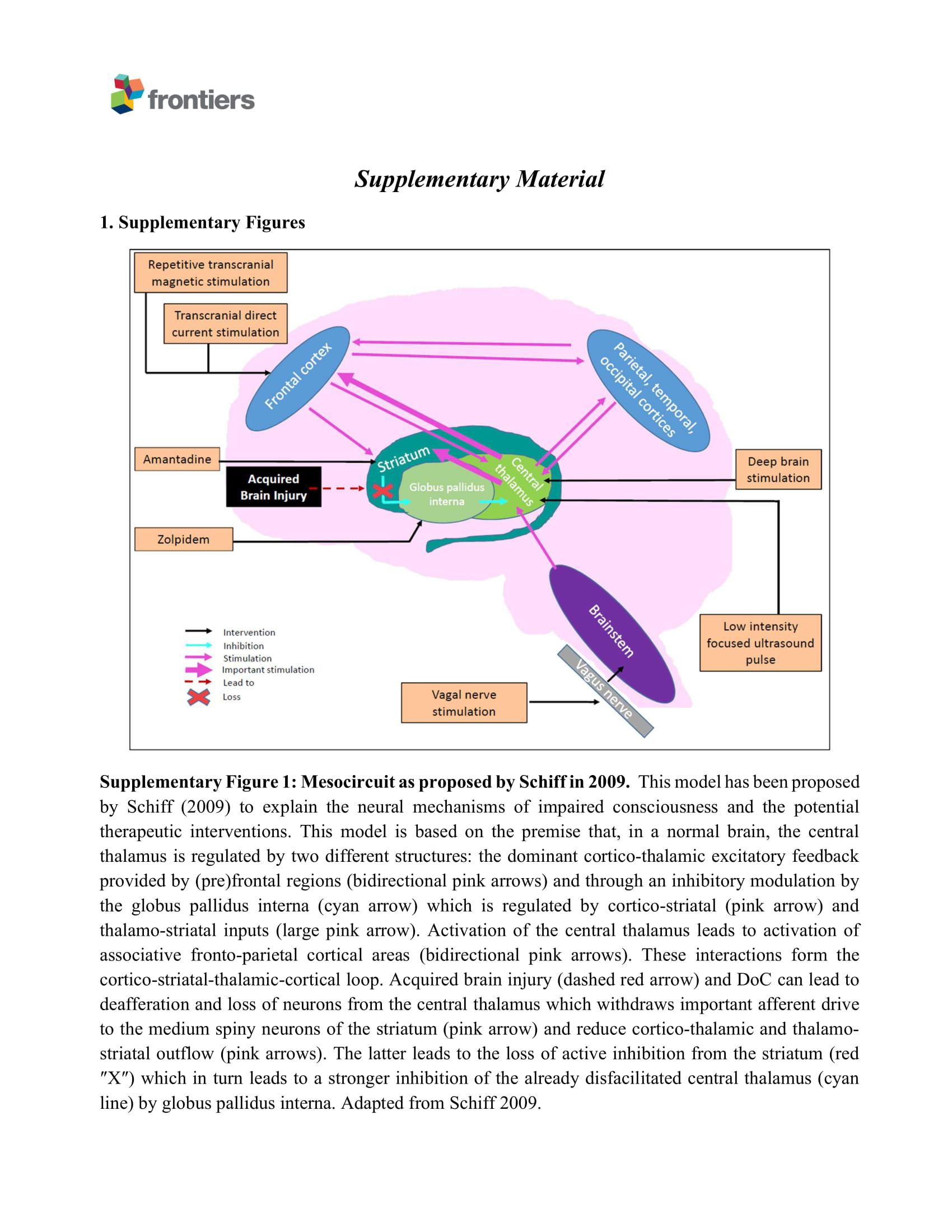

Supplement: Supplementary file 1 [file Image_1.JPEG]
